# 138. Impact of Emergency Department Rapid Viral Respiratory Multiplex PCR Testing on Antimicrobial Prescribing

**DOI:** 10.1093/ofid/ofad500.211

**Published:** 2023-11-27

**Authors:** Cassandra Falk, Lisa E Dumkow, Abigail Geyer, Nnaemeka Egwuatu, Jennifer Langholz, Kyle Schmidt, Andrew Jameson

**Affiliations:** Trinity Health Grand Rapids, Grand Rapids, Michigan; Trinity Health Grand Rapids, Grand Rapids, Michigan; Trinity Health Grand Rapids, Grand Rapids, Michigan; Trinity Health Grand Rapids, Grand Rapids, Michigan; Trinity Health Grand Rapids, Grand Rapids, Michigan; Ferris State University College of Pharmacy, Grand Rapids, Michigan; Trinity Health Grand Rapids, Grand Rapids, Michigan

## Abstract

**Background:**

Rapid multiplex polymerase chain reaction (mPCR) point-of-care tests detect a variety of viral respiratory pathogens. The impact of mPCR results on antibiotic prescribing in the ED or downstream is not well understood. The purpose of this study was to assess the impact of positive viral mPCR results on antibiotic prescribing for patients presenting with respiratory symptomsin the ED.

**Methods:**

This retrospective cohort study included adult patients presenting to the ED with respiratory symptoms and a positive rapid upper RTI mPCR for a non-SARs-CoV-2 viral pathogen between 11/1/2021 and 10/31/2022. The primary objective was to describe the proportion of patients with appropriate antibiotic management within 24 hours of positive mPCR result. Appropriate management was defined as patients without risk factors for bacterial co-infection who did not receive or continue antibiotic therapy. Those with co-infection risk factors prescribed antibiotic therapy were also considered appropriate. This was determined through independent pharmacist and ID physician review. Secondary objectives compared patient outcomes of those appropriately vs inappropriately managed and admitted vs discharged home from ED. Risk factors for inappropriate antibiotic decisions were also evaluated using logistic regression.

**Results:**

250 patients were included, 206 (82.4%) with appropriate management and 44 (17.6%) with inappropriate management. 164 (65.5%) patients were not prescribed empiric or definitive antibiotics. 39 (15.6%) patients empirically received antibiotics prior to mPCR; 9 (23%) were discontinued following positive viral result. Patients discharged home from the ED were more likely to have appropriate antibiotic management than those admitted (87.5% vs 77.7%, p=0.042). Patients with inappropriate antibiotic management were more likely to be readmitted to the hospital within 30 days (14% vs 3.5%, p=0.005). Purulent sputum (OR 3.61 [95% CI 1.64-8]) and unilobar infiltrate (OR 3.03 [1.22-7.58]) were independent risk factors for inappropriate antibiotic management.

**Conclusion:**

Rapid viral RTI mPCR testing in the ED resulted in a high level of appropriate antibiotic management, with most patients not being prescribed antibiotics.

**Disclosures:**

**All Authors**: No reported disclosures

